# Editorial: New Perspectives and Methodologies in the Diagnosis and Rehabilitation of Aphasia

**DOI:** 10.3390/brainsci11111508

**Published:** 2021-11-14

**Authors:** Francesca Pisano, Paola Marangolo

**Affiliations:** Department of Humanities Studies, University Federico II, 80133 Naples, Italy; pisanofrancescapsico@gmail.com

Aphasia is a highly disabling acquired language disorder, usually caused by left-lateralized brain damage. Due to its profound impact on a patient’s life, clinicians and speech-pathologists are continuously faced with the arduous challenge of enhancing aphasia recovery patterns and, subsequently, long-term outcomes in these individuals. However, even if traditional linguistic-based therapies have been proved to induce adequate clinical improvement, a large percentage of patients are left with some degree of language impairment. Thus, new approaches to common speech therapies are urgently needed in order to maximize recovery from aphasia. In recent decades, scientific advancements in language conceptualization and the progress of new technologies have made new tools available for professional therapists and educators. Although some of these approaches have shown promise, the results are not conclusive, and their interpretation is further compounded by the heterogeneity of study characteristics. In this Special Issue, a range of exciting contributions (10 articles and 2 reviews) which provide novel methods for the diagnosis and treatment of aphasia in clinical practice are reported.

“Are People with Aphasia (PWA) Involved in the Creation of Quality of Life and Aphasia Impacted-Related Questionnaires? A Scoping Review” by Charalambous and colleagues [1], the opening contribution to the Special Issue, provides an overview of the existing gap in the literature on the involvement of people with aphasia (PWA) as co-researchers, stakeholders, and patient partners for the construction of quality of life (QoL) and aphasia impact-related questionnaires (AIR-Qs). This scoping review, based on the guidelines of Preferred Reporting Items for Systematic Reviews and Meta-Analyses extension for Scoping Reviews (PRISMA-ScR), reports that only four works involved PWA in AIR-Qs, and none in QoL creation. In the selected AIR-Qs studies, in which the conceptual frameworks and the gold-standard tools were clearly described, PWA are engaged mainly as consultants or advisors. The authors highlight the need for ecological methodological approaches which should involve patients at each stage of the questionnaires’ construction in order to implement the reliability of the clinical assessment.

In addition to the need to implement self-report questionnaires involving the active participation of PWA, this collection contains three papers on the standardization of performance-based tests, which are relevant for the diagnosis and the objective evaluation not only of the linguistic components (verbal and/or no-verbal), but also of the cognitive factors that may impact the language difficulties experienced by PWA.

Ditges and colleagues’ article, “German Language Adaptation of the NAVS (NAVS-G) and of the NAT (NAT-G): Testing Grammar in Aphasia” [2], focuses on the development of the Northwestern Assessment of Verbs and Sentences (NAVS-G) and the Northwestern Anagram Test (NAT-G), two novel and flexible test batteries for testing syntactic complexity (including verb production, processing of simple and complex sentences) in native German-speaking left-hemisphere stroke patients. To investigate the effects of verb argument structure complexity and the canonicity of sentences, NAVS-G and NAT-G were administered to 27 healthy subjects, 15 right-hemispheric stroke patients without aphasia and 15 left-hemispheric stroke patients with mild aphasia; also taken into consideration were the patients’ demographic variables (age and educational level) and stroke-related factors (such as lesion location, size, etiology, and stroke severity). The results showed the reliability and sensitivity of NAVS-G and the NAT-G in analyzing the syntactic performance of the different participants, revealing their effectiveness in diagnostic evaluation.

“Nonverbal Semantics Test (NVST)-A Novel Diagnostic Tool to Assess Semantic Processing Deficits: Application to Persons with Aphasia after Cerebrovascular Accident” by Hogrefe and collaborators [3] is another important contribution concerning the standardization of an instrument for the clinical assessment of non-verbal semantic skills. After an introduction which highlights the importance of testing semantic cognition in PWA and the lack of non-verbal semantic tests in the aphasia field, which are essential to diagnose patients with severe aphasia, the authors investigated: (1) the performance of the NVST on fifty-one people with aphasia (mild, moderate, and severe) in the acute phase and possible correlations among the different subtests of the NVST (semantic sorting, drawing, and pantomime); (2) the relationship between the NVST subtests and the neurolinguistic measures of the Aachen Aphasia Test (AAT). Overall, the data showed that the NVST is able to discriminate the performance of participants with varying degrees of severity, and that the different subtests correlate among each other and with the AAT tasks.

In line with these studies, Robinson et al.’s paper, “A Brief Executive Language Screen for Frontal Aphasia” [4], focuses on the development of an abbreviated aphasia screening tool designed to assess the executive components of language generation in a cohort of one hundred and thirty-eight stroke patients matched with one hundred and eight controls. Based on contemporary theoretical models which posit that different cognitive processes contribute to spoken language generation, the Brief Executive Language Screen (BELS) assesses propositional language (spontaneous speech), nominal language functions (repetition, comprehension, naming, and reading), and the role of executive components in the conceptualization phase of language generation. In the Materials section, detailed descriptions of the 11 BELS subscales are reported. In summary, the results indicated that the BELS has good internal consistency, discriminant validity, and sensitivity. It is therefore a valid tool for identifying executive language deficits which might be crucial for explaining some of the symptoms observed in PWA, thus, implementing the clinical diagnosis.

In addition to the effectiveness of the evaluation tools outlined above, this Special Issue includes a number of evidence-based studies using new and cutting-edge theoretical approaches to find new solutions to improve the language recovery process.

“Main Concept, Sequencing, and Story Grammar Analyses of Cinderella Narratives in a Large Sample of Persons with Aphasia” by Richardson and colleagues [5] illustrates how a multi-level analytical approach, characterized by a main concept, sequence, and story grammar (MSSG) is useful for understanding discourse informativeness and macrostructure in PWA. Tested on a large sample of 238 PWA and 95 non-brain-injured people (PNBI), this approach reports different profiles between PWA and PNBI for all MSSG variables. Differences are presented for each aphasia subtype, even on mild aphasics. The authors also provide readers with normative information for MSSG categorization that might be applied to their own patients.

In their paper “Creating a Theoretical Framework to Underpin Discourse Assessment and Intervention in Aphasia”, Dipper et al. [6] propose a new unified theoretical framework, Linguistic Underpinnings of Narrative in Aphasia (LUNA). After reviewing the literature, the authors show that none of the existing theoretical perspectives describe all of the categories of spoken discourse in sufficient detail, nor fully address the relationship of one category to another. Given that aphasia affects discourse in a range of ways (i.e., the language elements that speakers use, the information communicated, and the structure of discourse information), the LUNA approach, which is comprised of four subtests (i.e., linguistic, propositional, macrostructure planning and pragmatic components), can be used to analyze spoken language discourse in PWA in order to achieve a complete diagnosis, useful in research and clinical practice.

To identify a new model which investigates the functional integrity of brain regions in chronic aphasia, Abbott and coworkers, in their paper “Defining Hypoperfusion in Chronic Aphasia: An Individualized Thresholding Approach” [7], examine how an individualized thresholding approach might quantify the extent of hypoperfused tissue in the perilesional regions. The authors investigate the relationship between the amount of hypoperfusion in language areas and the language behaviors of six individuals with aphasia post-stroke. In contrast to a standard cutoff model, the authors show that this individualized metric more accurately identifies functionally impaired areas and the resulting language disorders. Although time-consuming, it allows to better represent the variability in cerebral blood flow (CBF) patterns between individuals and explain post-stroke behavioral changes, both aspects being important for the design and implementation of rehabilitation interventions.

A key issue in aphasia rehabilitation is the need to implement traditional language treatments with new approaches that are effective and safe. The review by Picano et al., “Adjunctive Approaches to Aphasia Rehabilitation: A Review on Efficacy and Safety” [8], examines the approaches most commonly used in recent years (pharmacology, virtual reality, and transcranial direct current stimulation) in the field of aphasia, which go beyond the classical view of language representation. Indeed, all of these approaches rely on the hypothesis that the language system is not modularized into specific language areas but is considered as a widely distributed network across the brain. The strengths and weaknesses of each approach are critically examined, emphasizing the urgency of promoting large randomized clinical trials in order to understand which is the best solution and, more importantly, which patients are likely to benefit.

Regarding the effectiveness of neuromodulation techniques, such as transcranial direct current stimulation (tDCS), Themistocleous and collaborators in “Effects of tDCS on Sound Duration in Patients with Apraxia of Speech in Primary Progressive Aphasia” [9], investigate whether tDCS over the left inferior frontal gyrus (IFG) coupled with speech production therapy reduce apraxia of speech (AOS) symptoms in patients with non-fluent primary progressive aphasia (nfvPPA/AOS). To answer their research questions, in a randomized double-blind design, eight nfvPPA/AOS underwent five days of tDCS over three weeks with concomitant treatment for their articulatory deficits in two different conditions: anodal and sham. In all patients, language measures were collected before (T0), at the end (T15) and two months after the end of treatment (FU). The authors used sound duration as a measure of AOS symptoms and reduced sound duration as an improvement of speech production in these patients. Results showed that sound duration significantly decreased after anodal tDCS compared to the sham, maintaining this advantage up to two months post-intervention. Instead, the generalization effect was only reported at the end of the intervention. This evidence suggests that tDCS has the potential to improve speech production, even in neurodegenerative disorders such as nfvPPA.

Given the absence of a standard protocol for the application of tDCS, in “Extended fMRI-Guided Anodal and Cathodal Transcranial Direct Current Stimulation Targeting Perilesional Areas in Post-Stroke Aphasia: A Pilot Randomized Clinical Trial” by Cherney and colleagues [10], the safety and efficacy of fMRI-guided tDCS combined with speech language therapy (SLT) on a cortical activation map and language abilities across an extended treatment period are investigated. In a single-blind randomized trial, twelve persons with chronic non-fluent aphasia were divided into three groups: (1) anodal tDCS + SLT, (2) cathodal tDCS + SLT, and (3) sham conditions, characterized by language treatment only. Greater improvements of linguistic skills after active and cathodal stimulations with respect to the sham were found. The authors confirm a higher efficacy of language treatment associated with tDCS, but the innovative finding is that no adverse events occurred during the 30 treatment sessions, demonstrating that tDCS (1 mA 13 min) is safe and also well-tolerated over a long period of time.

This collection also features a study using diffusion tensor imaging (DTI), a neuroimaging tool which assesses white matter integrity to predict the severity of language disorders in patients with post-stroke aphasia. In particular, Lee and collaborators’ work, “Prediction of Aphasia Severity in Patients with Stroke Using Diffusion Tensor Imaging” [11], focus on the classification of aphasia severity through the Western Aphasia Battery (WAB), determining the optimal cutoff scores for each language-related white matter (LRWM) fiber that relies on ventral and dorsal pathways. A detailed argumentation of the results on the correlation between LRWM and the scores on the different subtests of the WAB is presented.

Finally, of interest, is the contribution “Sentence Recall in Latent and Anomic Aphasia: An Exploratory Study of Semantics and Syntax” of Salis and colleagues [12], which addresses the effect of semantic and syntactic plausibility in sentence recall in people with anomic and latent aphasia using accuracy and real-time speech measures. In contrast to accuracy, which appears not to distinguish performance between latent aphasics and controls, results showed that temporal measures are sensitive in discriminating performance among the participants. The authors explain the slower recall of implausible sentences referring to the conceptual theory of memory (STM), which provides important insights into the effectiveness of language treatments in clinical practice.

Overall, the twelve contributions which are part of this Special Issue provide novel data, new materials, and fruitful thoughts from experts in the field, which should be taken into serious consideration in clinical practice with PWA. The future challenge will be to replicate the effectiveness of these methods and/or tools on large samples of PWA and to include longitudinal follow-ups in order to further validate the reliability of these measurements. These precautions will have fundamental implications for improving the diagnosis of aphasic symptoms and, thus, the implementation of new rehabilitation protocols.

## Data Availability

Data sharing not applicable.
